# Supplementary material for: Ratioing the President: An exploration of public engagement with Obama and Trump on Twitter
Source: PLoS One. 2021 Apr 14;16(4):e0248880. doi: 10.1371/journal.pone.0248880 (PMC8046224; doi:10.1371/journal.pone.0248880)
Supplement: S1 Appendix — (PDF) [file pone.0248880.s001.pdf]

S1 Appendix.

Ternary histogram snapshots

Snapshots of ternary histograms are another way of investigating the characteristic time scale described in Sec. 3.4 and presented in Fig. 6. Here, we calculate the ternary ratios at logarithmically spaced intervals after a tweet is authored (this is in contrast to the stabilized or 'final' ratio values in Fig. 3).

S1 Fig. and S2 Fig. show histograms of the ternary ratio values for Obama and Trump tweets at logarithmically spaced time intervals after a tweet is authored. For both presidents there is a greater spread in ternary ratio values immediately after a tweet is authored, with ratios tending to stabilize in the hours and days after a tweet is released. For Trump's tweets, the tendency for ternary ratio values to have a greater reply component is seen throughout the progression of the snapshots. Compared to Obama, Trump tends to have greater spread in the distribution of ternary ratios for each time interval. These results are consistent with our understanding of activity stabilization occurring roughly 1 day after a tweet is authored (as shown in Fig. 7).
