# Supplementary material for: Ratioing the President: An exploration of public engagement with Obama and Trump on Twitter
Source: PLoS One. 2021 Apr 14;16(4):e0248880. doi: 10.1371/journal.pone.0248880 (PMC8046224; doi:10.1371/journal.pone.0248880)
Supplement: S2 Appendix — (PDF) [file pone.0248880.s002.pdf]

S2 Appendix.

Brief note on bots

In the main body, we discuss the likely influence of bots in the response activity time series. While we do not address this topic in-depth at any point in the present work, we reviewed accounts that retweet or reply to one of two Trump and two Obama tweets in order to gain a preliminary understanding of the volume of bot activity.

Bot detection is a challenging and open-ended task. We use Botometer (`https://botometer.osome.iu.edu/`) to score the likelihood that accounts are bots. We randomly select 200 unique users who retweeted highly-liked Trump and Obama tweets. We also randomly select 200 unique users who replied to the same tweets, presenting these results separately. We then run these users through Botometer and report the conditional probability ("cap") that users with a similar score are bots.

For the 200 retweets of Obama tweets, 2.3% had conditional probability scores greater than or equal to 0.8 ($\text{cap} \geq 0.8$). For the 200 retweets of Trump tweets we found that 9.5% of tweets had cap values greater than or equal to 0.8.

For the 200 replies, 16.9% and 8.0% of the Obama and Trump replies, respectively, had conditional probability scores greater than or equal to 0.8.

These results are initial estimates of the overall prevalence of bots in the response activity data. It provides a sense for the order of magnitude of bot activity that may truly be occurring. There is ample room for future research on a larger sample and with more developed methods.

The two Trump tweets examined: `https://twitter.com/realDonaldTrump/status/1157345692517634049` and `https://twitter.com/realDonaldTrump/status/881503147168071680`.

The two Obama tweets examined: `https://twitter.com/BarackObama/status/896523232098078720` and `https://twitter.com/BarackObama/status/1221552460768202756`.
