# Supplementary figures and images for: Ratioing the President: An exploration of public engagement with Obama and Trump on Twitter
Source: PLoS One. 2021 Apr 14;16(4):e0248880. doi: 10.1371/journal.pone.0248880 (PMC8046224; doi:10.1371/journal.pone.0248880)

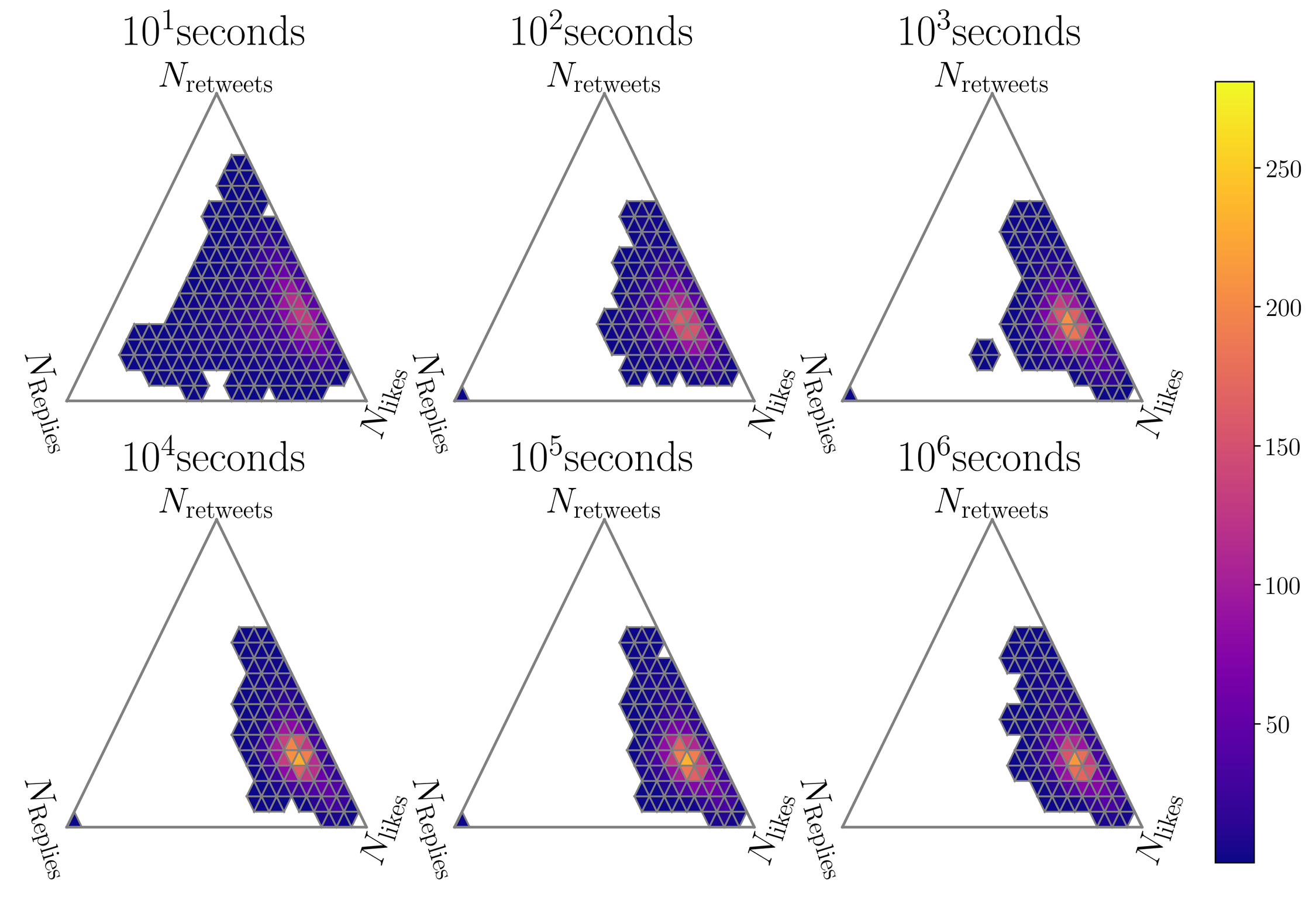

Supplement: S1 Fig — Observations are recorded at logarithmically spaced intervals after the release of the original tweet. Included here are 3,015 tweets from the period of time after Trump’s declaration of candidacy on June 16, 2015. Whereas Fig 3 shows a final ratio value for tweets over distinct political periods, here we show how ratios unfold over the life of each tweet. This is serves as a snapshot of the ternary ratio time series presented in Fig 4. Obama’s tweets tend to receive a greater volume of likes and retweets relative to replies, and this ratio is often maintained throughout the response timeline. (TIF) [file pone.0248880.s003.tif]

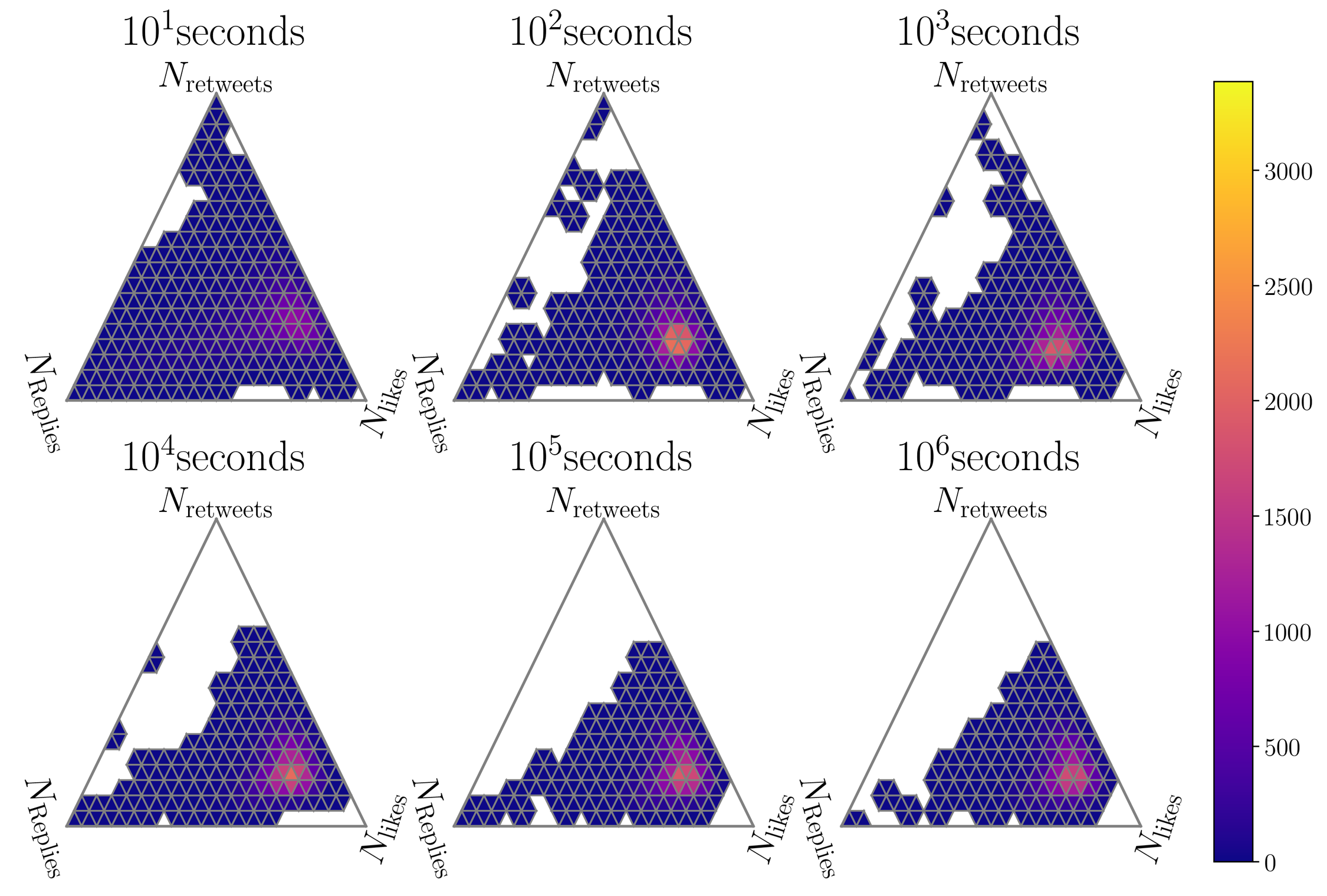

Supplement: S2 Fig — Observations are recorded at logarithmically spaced intervals after the release of the original tweet. Included here are 16,708 tweets from the period of time after Trump’s declaration of candidacy on June 16, 2015. Whereas Fig 3 shows a final ratio value for tweets over distinct political periods, here we show how ratios unfold over the life of each tweet. This is serves as a snapshot of the ternary ratio time series presented in Fig 4. We can see the greater variation in ternary ratio values compared to the snapshots for Obama (Fig 4). There is also a greater tendency for tweets to have higher reply counts when compared to the Obama snapshots. (TIF) [file pone.0248880.s004.tif]

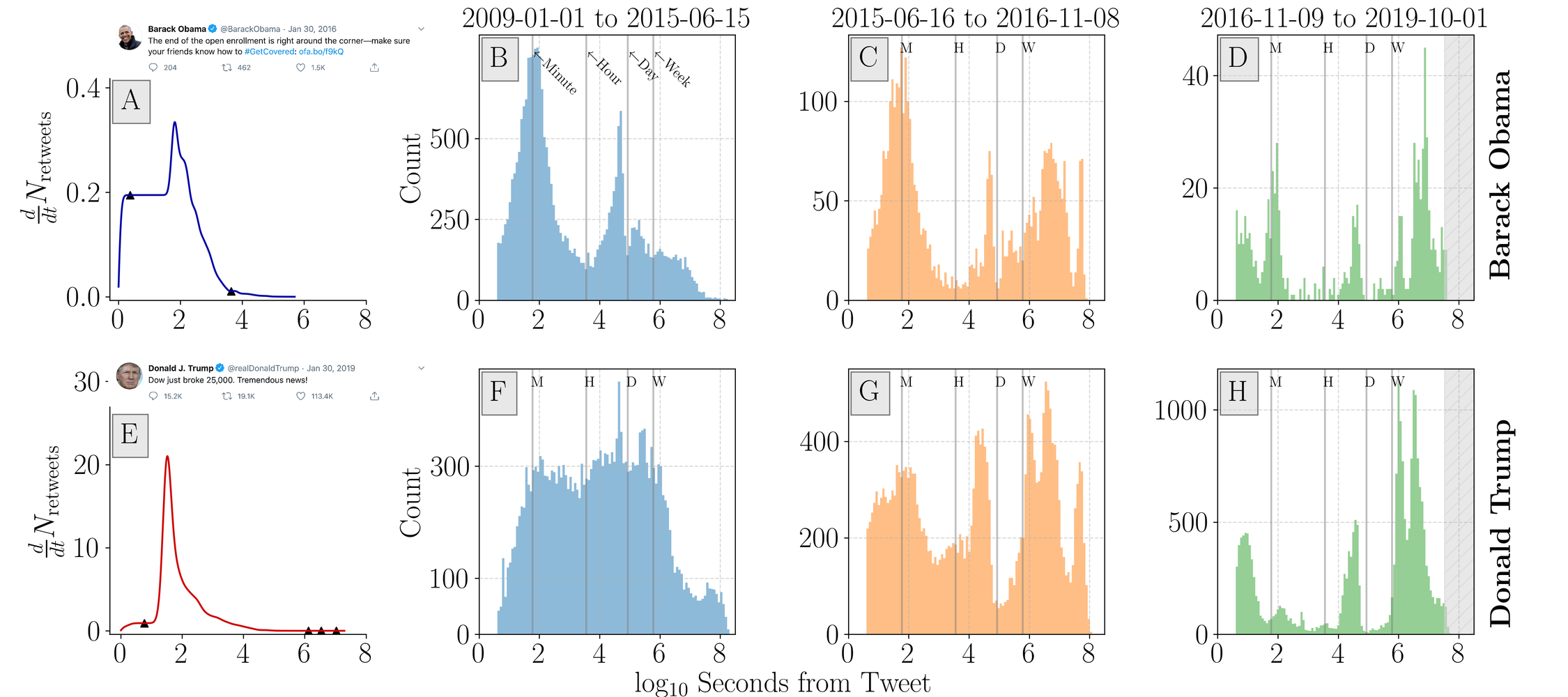

Supplement: S3 Fig — Here we switch the criteria from Fig 6 in order to show how our choice affects the results for the characteristic time scale. For the results in this figure we show points where the second derivative of the retweets activity time series goes from negative to positive. A and E: Example cumulative retweet time series and inverse-inflection points (solid triangles) for Obama and Trump tweets. Histograms show the distribution of inverse-inflection points across all tweets binned by time periods before (B and F), during (C and G), and after (D and H) the 2016 US presidential election campaign. The January 1st 2009 to June 15th 2015 period for Trump (F) contains inflection point counts that are largely reflective of low initial activity and high(er) late activity (months or years later) leading to unusually high values for seconds to first inflection point (>108 seconds). For Obama’s and Trump’s time in office, tweets experience inflection points around 1-minute and 1-day after the tweet is authored—indicating characteristic time-scales of activity waning. Direct links for Obama tweet (A): https://twitter.com/BarackObama/status/693571153336496128 and Trump tweet (E): https://twitter.com/realDonaldTrump/status/1090729920760893441. Screenshots were collected on May 28, 2020. (TIF) [file pone.0248880.s005.tif]

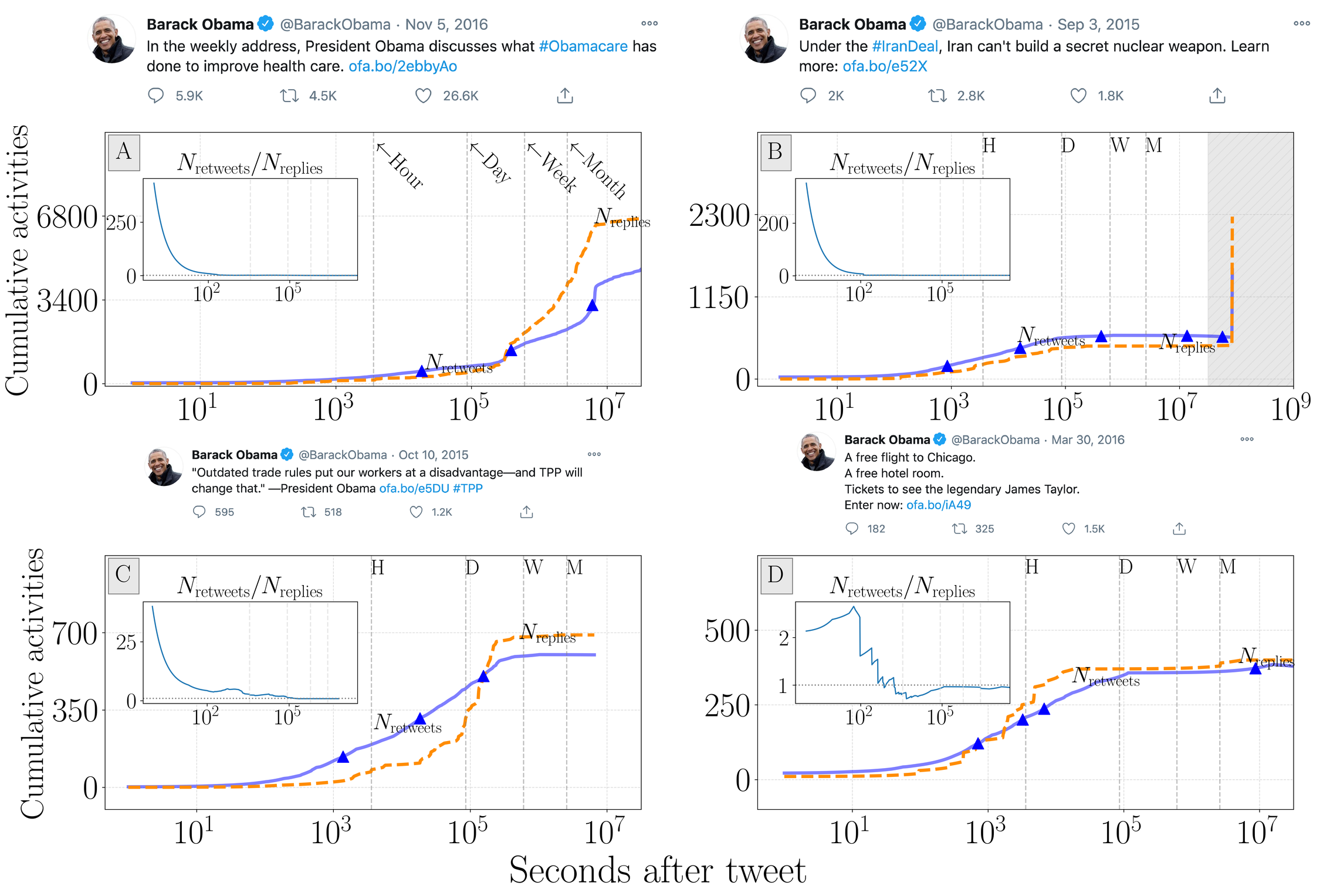

Supplement: S4 Fig — Here we show the instances where we observed Obama’s tweets garnering more replies than retweets. For the tweets in panels (B, D and G) the response activities later changed to reflect fewer replies than retweets. This behavior is consistent with what we have seen in other cases, with activity shifts either owing to authors deleting the activity or responding-accounts being deleted. Here it seems Obama’s tweets are a combination of controversial (the Iran nuclear deal) and engagement-seeking (soliciting entries in a contest to meet the Obama). Direct links to tweets for panel (A): https://twitter.com/BarackObama/status/733055491664797696, panel (B): https://twitter.com/BarackObama/status/639553864006430721, panel (C): https://twitter.com/BarackObama/status/794926969829920768, panel (D): https://twitter.com/BarackObama/status/666396723191808000, panel (E): https://twitter.com/BarackObama/status/652914197454561281, panel (F): https://twitter.com/BarackObama/status/732691885139984384, and panel (G): https://twitter.com/BarackObama/status/715194440487309312. Tweet screenshots were collected on November 15, 2020. (TIF) [file pone.0248880.s006.tif]
